# A Histological Analysis and Detection of Complement Regulatory Protein CD55 in SARS-CoV-2 Infected Lungs

**DOI:** 10.3390/life14091058

**Published:** 2024-08-23

**Authors:** Sandeep Silawal, Clemens Gögele, Petr Pelikan, Christian Werner, Georgia Levidou, Raman Mahato, Gundula Schulze-Tanzil

**Affiliations:** 1 Institute of Anatomy and Cell Biology, Paracelsus Medical University, Nuremberg and Salzburg, General Hospital Nuremberg, Prof. Ernst Nathan Str. 1, 90419 Nuremberg, Germany; clemens.goegele@pmu.ac.at (C.G.); c.werner@pmu.ac.at (C.W.); gundula.schulze@pmu.ac.at (G.S.-T.); 2 Institute for Pathology, Paracelsus Medical University, Nuremberg, General Hospital, Prof. Ernst Nathan Str. 1, 90419 Nuremberg, Germany; petr.pelikan@klinikum-nuernberg.de (P.P.); georgia.levidou@klinikum-nuernberg.de (G.L.); 3 Department of Emergency and Intensive Care Medicine, Klinikum Ernst von Bergmann, Charlottenstraße 72, 14467 Potsdam, Germany; raman.mahato@klinikumevb.de

**Keywords:** ARDS, SARS-CoV-2, CD55, lungs, histology, immunofluorescence

## Abstract

Background: A complement imbalance in lung alveolar tissue can play a deteriorating role in COVID-19, leading to acute respiratory distress syndrome (ARDS). CD55 is a transmembrane glycoprotein that inhibits the activation of the complement system at the intermediate cascade level, blocking the activity of the C3 convertase. Objective: In our study, lung specimens from COVID-19 and ARDS-positive COVID+/ARDS+ patients were compared with COVID-19 and ARDS-negative COVID–/ARDS– as well as COVID–/ARDS+ patients. Methods: Histochemical staining and immunolabeling of CD55 protein were performed. Results: The COVID–/ARDS– specimen showed higher expression and homogeneous distribution of glycosaminoglycans as well as compactly arranged elastic and collagen fibers of the alveolar walls in comparison to ARDS-affected lungs. In addition, COVID–/ARDS– lung tissues revealed stronger and homogenously distributed CD55 expression on the alveolar walls in comparison to the disrupted COVID–/ARDS+ lung tissues. Conclusions: Even though the collapse of the alveolar linings and the accumulation of cellular components in the alveolar spaces were characteristic of COVID+/ARDS+ lung tissues, evaluating CD55 expression could be relevant to understand its relation to the disease. Furthermore, targeting CD55 upregulation as a potential therapy could be an option for post-infectious complications of COVID-19 and other inflammatory lung diseases in the future.

## 1. Introduction

The novel virus, 2019-nCoV (SARS-CoV-2), is the seventh coronavirus known to infect humans. As HCoV-229E, HCoV-OC43, HCoV-NL63, HCoV-HKU1 infect mainly the upper respiratory tract causing mild cold and flu-like symptoms, the highly pathogenic SARS-CoV (2002–2003), MERS-CoV (2012) and SARS-CoV-2 (2019) infect the lower respiratory tract of immunocompetent individuals causing a severe acute respiratory syndrome, a major complication of SARS-CoV-2 infections [1,2,3]. A SARS-CoV-2-related infection leads to the disease, namely COVID-19, which is associated with a mild course in 81%, a severe course in 14%, but a critical course in 5% with high mortality [4]. In an acute-phase reaction, there is an increase in the cytokines such as tumor necrosis factor α, interleukin (IL)-1β, IL-8, macrophage chemoattractant protein 1, etc., in the early period, followed by a further sustained increase in IL-6 [5]. Severe COVID-19 is characterized by an increased production of cytokines, a so-called “cytokine storm” [6]. Such cytokine storm in patients with SARS-CoV-2 infections leads to persistent activation of immune cells, potentially resulting in acute respiratory distress syndrome (ARDS), sepsis with multiple organ failure, and ultimately death [7,8].

In every stage of the disease, the innate immune system is always in action with one of its primary tools, the complement system [9]. The classical, lectin, and alternative pathways generally initiate the complement cascade, resulting in three major outputs, i.e., opsonization via C3b, inflammation via generation of anaphylatoxins C3a, C4a and C5a or osmotic lysis of the microbial entities or damaged cells generating membrane attacking complex (MAC). The anaphylatoxins C3a and C5a bind specifically to their receptors C3aR and C5aR1 or C5aR2 and promote chemotaxis and regulate effector functions of cells of both the innate and adaptive immune responses [9]. Not only the mobilization of the immune cells into the lungs but also an active trigger of other cytokines through these anaphylatoxins is promoted. An increase in soluble C5a levels has been described in serum and that of C5aR1 expression in blood and pulmonary myeloid cells in severe cases, suggesting a role for the C5a–C5aR1 axis in the pathophysiology of ARDS in COVID-19 patients [10]. Therefore, the inhibition of the complement and the anaphylatoxins has been postulated to be a potential COVID-19 therapeutic intervention [11,12,13]. Physiologically, soluble as well as membrane attached complement regulatory proteins dampen the hyperinflammatory activity of the complement system. In 1995, Varsano et al. examined tissue from the human respiratory system from the nose to the alveoli for the expression of membrane-bound complement–regulatory proteins [14]. This research group was able to detect CD46, CD55, and CD59 but not CD35 in the epithelial cells of the airways. One of these regulatory proteins, namely CD55, is a transmembrane glycoprotein that inhibits the activation of the complement system at the intermediate cascade level, blocking the activity of the C3 convertase [15]. A significantly higher CD55 expression in blood monocytes of COVID-19 patients was observed compared to healthy controls [16]. Pandya et al. illustrated the down-regulation of CD55 in hypoxic mouse lungs (in vivo) as well as in small-airway epithelial cells (in vitro) incubated in a 1% O_2_ hypoxic chamber [17]. There could be a possible discrepancy in CD55 expression in hypoxic lung tissue in comparison to the peripheral blood cells in COVID-19 patients. The role of complement therapy in COVID-19 and other respiratory virus diseases has been a matter of discussion in recent years [18,19,20]. CD55 is considered to be a positive regulator when it comes to tumorigenesis and malaria infection, whereas a negative regulator of the CHAPLE (CD55 deficiency with hyper-activation of complement, angiopathic thrombosis, and severe protein-losing enteropathy) syndrome, paroxysmal nocturnal hemoglobinuria, multiple sclerosis, and autoimmune diseases [20]. Our study aims to highlight the histopathological features as well as CD55 protein expression in the COVID-19-related or COVID-19 non-related damaged lung tissue in comparison to non-damaged lung tissue.

## 2. Materials and Methods

The lung specimens were derived from the Institute for Pathology, Paracelsus Medical University, Nuremberg, General Hospital. Eight tissue samples were obtained as part of the pathological autopsy, and four samples were obtained from the tumor-free area of the partial lung resection. These four samples were used as the control group. The paraffin embedding of the respective tissues was carried out at the Institute of Pathology. The pathological autopsy was performed 1–3 days post-mortem. The specimens were extracted and fixed in 4% paraformaldehyde (PFA, ThermoFisher Scientific, Darmstadt, Germany) for at least 3 weeks before they were processed further. Table 1 illustrates the lung specimens included in this investigation. Category 1 represents our control group with lung specimens from patients without COVID-19 as well as ARDS (COVID–/ARDS–), category 2 represents the group with lung specimens from patients with COVID-19 as well as ARDS (COVID+/ARDS+) and category 3 represents the group with lung specimens from patients without COVID-19, but ARDS (COVID–/ARDS+) (Table 1). The time frame (year) where the lung tissues were collected for the study are: Category 1 (2012–2023), Category 2 (2020–2022), Category 3 (2020–2023). The research was approved by the Institutional Review Board of Klinikum Nuremberg, Germany, approval number SZ_FP_089.20-KNMS.

### 2.1. Histological Staining

The PFA-fixated lung tissues were cut and dehydrated in ascending ethanol series before they were embedded in paraffin. The tissue was sliced using a microtome (ThermoFisher Scientific, Darmstadt, Germany) to prepare 5 µm thick tissue slides. The slides were then baked at 60 °C overnight. After this process, the slides were treated with xylene (Carl Roth GmbH, Karlsruhe, Germany) to remove paraffin from the slides. After the rehydration process with descending ethanol series (Carl Roth GmbH, Germany), various staining methods mentioned below were performed consecutively. Finally, all the histological images (except 2.1.5.) were assessed using digital microscopy (PreciPoint Fritz, Garching bei München, Germany).

#### 2.1.1. Hematoxylin Eosin Staining

After rehydration, the slides were rinsed in distilled water and then immersed in Harry’s hematoxylin (Carl Roth GmbH, Germany) for four minutes. Subsequently, the slides were dipped in tap water and thereby blued. Thereafter, the slides were rinsed with 96% ethanol and immersed in Eosin Y (Carl Roth GmbH, Germany) for 1.5 min. For dehydration, the slides were dipped in ascending ethanol solutions again. Finally, the slides were immersed in xylene for five minutes, then covered with entellan (Merck KGaA, Darmstadt, Germany) and dried at room temperature (RT) until the next day.

#### 2.1.2. Alcian Blue Staining

After rehydration (Section 2.1), the slides were rinsed in distilled water and rinsed in 1% acetic acid (Carl Roth GmbH, Germany) for three minutes and subsequently immersed in a 1% alcian blue solution (Merck KGaA, Darmstadt, Germany), at pH 2.1 for 15 min. Thereafter, the slides were rinsed with 3% acetic acid and then with distilled water before being immersed in a nuclear-fast red aluminum sulfate solution (Carl Roth GmbH, Germany) for five minutes. Immediately afterward, the slides were rinsed in distilled water for two minutes. The slides were dehydrated in the ascending ethanol dilutions for two minutes. Finally, the slides were immersed in xylene for five minutes and then covered with entellan.

#### 2.1.3. Elastica van Gieson Staining

After rehydration (Section 2.1), the slides were immersed in Weigert’s hematoxylin (Carl Roth GmbH, Germany) for 5 min. Then, the slides were rinsed in tap water for 3 min before they were dipped in van Gieson solution (Morphisto GmbH, Offenbach am Main, Germany) for 1 min. The slides were rinsed in the ascending ethanol dilutions for two minutes for dehydration. Finally, the slides were immersed in xylene for 3 min and covered with entellan.

#### 2.1.4. Masson-Goldner Staining

After rehydration (Section 2.1), the slides were immersed in Weigert’s hematoxylin (Carl Roth GmbH, Germany) for 5 min. Then, the slides were rinsed in tap water for 10 min before dipping them in Goldner I, II, and III solutions (Carl Roth GmbH, Germany) for 5, 30, and 60 min, respectively. Next, 1% acetic acid was used for the rinsing process between the changes in the Goldner solutions. After the final dyeing process, the slides were rinsed in the ascending ethanol solution for dehydration. Finally, the slides were immersed in xylene for 20 min, covered with entellan, and dried at RT until the next day.

### 2.2. Immunofluorescence Staining

After rehydration (Section 2.1), the slides were rinsed in phosphate-buffered saline (PBS, Carl Roth GmbH, Germany). For antigen retrieval, the slides were treated in a pressure cooker (Rommelbacher ElektroHausgeräte GmbH, Dinkelsbühl, Germany) at 97 °C, pH 6, and steam pressure of 1 bar for 10 min. After the antigen retrieval procedure and cooling of the slides upto RT, the slides were first rinsed in PBS for 5 min before the primary antibody, CD55 (Antigen Affinity-purified Polyclonal Goat IgG, Catalog Number: AF2009, R&D systems, Minneapolis, MN, USA) [21] diluted in blocking solution was pipetted onto the slides and incubated at 4 °C overnight. The blocking solution contained 5% Donkey serum (Chemicon, Temecula, CA, USA), 0.1% Triton X-100 (Sigma-Aldrich, St. Louis, MO, USA) in Tris-buffered saline (TBS). The next day, the slides were first washed twice for 5 min. Immediately thereafter, the slides were incubated with the secondary antibody (Cyanine 3 (Cy3)-AffiniPure Donkey Anti-Goat IgG, Catalog Number: 705-165-147, Jackson ImmunoResearch Laboratories Inc., Pennsylvania, USA) dissolved in blocking solution for 1 h at RT. The cell nuclei were counterstained using 4′,6-diamidin-2-phenylindol-dihydrochlorid (DAPI, Catalog Number: D9542, Sigma-Aldrich, USA). Again, after 2 washes, the slides were mounted using Fluoromount G (SouthernBiotech, Birmingham, AL, USA). Three representative images per lung specimen (200x magnification) were taken using confocal microscopy (Leica TCS SPEII and DMi8, Leica Microsystems, Wetzlar, Germany). The intensity of the red signals in each image was measured with the 3D CLSM Leica software (Version 3.5.7.23225). The staining artifacts, if present, were excluded from the data curation manually. The mean intensity of the three replicates (i.e., images) per lung specimen was used in the statistics. These mean values were now divided in each category for the statistical analysis. Data are expressed as mean values in each category with standard deviation (SD). GraphPad Prism8 (GraphPad Software Inc., San Diego, CA, USA) was used to perform statistics and for the graphical representation. Shapiro-Wilk test was used for the analysis of the normal distribution of the results. Ordinary one-way was used, followed by Dunnett’s post hoc testing to compare the control group with each treatment group. The level of significance was set at *p*-values of ≤0.05 (*).

## 3. Results

### 3.1. Histological Evaluation of the Lung Specimens

**Figure 1:**
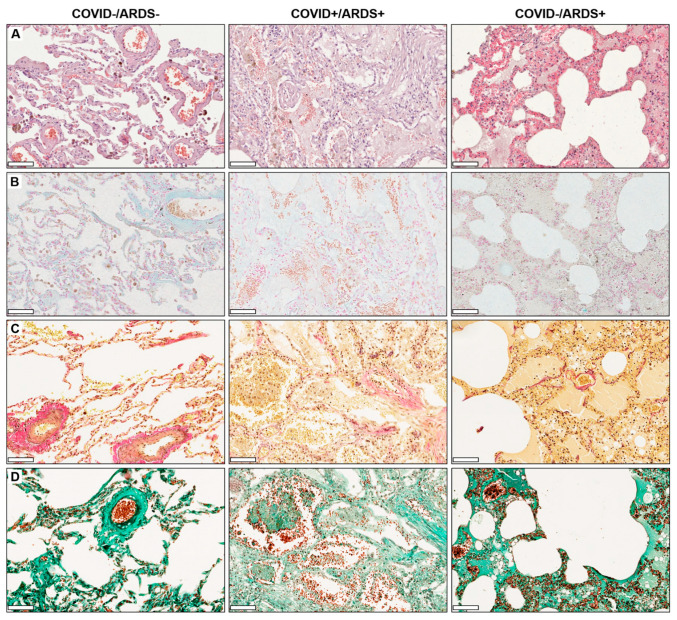
Representative images of lung tissue specimens with (**A**) hematoxylin eosin staining, (**B**) alcian blue staining, (**C**) Elastica van Gieson and (**D**) Masson-Goldner staining of COVID–/ARDS–, COVID+/ARDS+ and COVID–/ARDS+ patients. Scale bar 100 µm.

In the hematoxylin eosin-stained images (Figure 1A), COVID-/ARDS- lungs showed a regular alveolar space structure. Alveolar macrophages could be detected in the alveolar lumen. The alveolar spaces in the COVID+/ARDS+ lung specimens were filled with infiltrates composed of cellular debris and fibrinous material (Figure 1A). In addition to hemorrhage in the alveolar space, fibroproliferative response in the interstitium associated with abundant collagenous material deposition was observed. COVID–/ARDS+ showed a variety of characteristics with largely characterized fibroproliferative and/or edematous changes in the interstitium.

COVID–/ARDS– lung specimens in alcian blue staining (Figure 1B) showed a strong homogeneous expression of glycosaminoglycans (GAG) on the alveolar walls as well as the vessel walls. COVID+/ARDS+, as well as COVID–/ARDS+ lungs, displayed a weaker signal and a rather diffuse interstitial distribution. Accumulation of blood tissue was detected in the narrowed alveolar spaces in COVID+/ARDS+ lungs.

The fibrous alveolar wall integrity of the examined lung specimens was analyzed using Elastica van Gieson (Figure 1C) and Masson-Goldner (Figure 1D) staining. Both staining displayed that the integrity of the alveolar walls is maintained in the COVID–/ARDS– lungs. However, COVID+/ARDS+ lung specimens showed that the collagenous, as well as elastic fibrous tissue, has disintegrated drastically and displayed once again the poor integrity of the alveolar walls. In this staining, the alveolar spaces can be better detected, which are filled with cellular components embedded in a collagenous extracellular matrix, as shown via Masson-Goldner and Elastica van Gieson staining.

### 3.2. Immunofluorescence Staining of CD55 in the Lung Specimens

**Figure 2:**
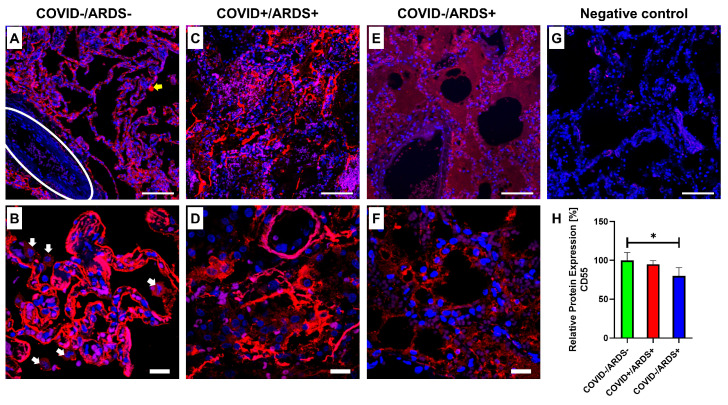
Representative overlay immunofluorescence images of lung tissues derived from COVID–/ARDS–, COVID+/ARDS+, and COVID–/ARDS+ patients in 200× (upper row: (**A**,**C**,**E**,**G**). Scalebar: 100 µm) and 630× (lower row: (**B**,**D**,**F**). Scalebar: 20 µm) magnification. Red (Cy3) = CD55, blue (DAPI) = cell nuclei. The primary antibody against CD55 was omitted in the negative control. (**A**) The blood vessel inside the lung tissues has been indicated by the oval lining. The yellow arrow indicates a staining artifact. The white arrows in image (**B**) depict alveolar macrophages with positive CD55 staining. (**H**) Graphic representation of relative CD55 protein fluorescence intensity measured in [COVID–/ARDS–, n = 4], [COVID+/ARDS+, n = 5] and [COVID–/ARDS+, n = 3], lung tissues. Mean with standard deviation (SD). The COVID–/ARDS– group has been normalized to 100. Repeated Measures one-way ANOVA using Dunnett’s multiple comparisons (*). * = *p* ≤ 0.05.

The semi-quantitative evaluation of the CD55 protein expression analyzing the fluorescence intensity of the images demonstrated that CD55 expression in the alveoli lining is significantly reduced in the COVID-/ARDS+ lungs compared to the control group, i.e., COVID–/ARDS– lungs (Figure 2H). A non-significant decrease in the CD55 signal intensity could be detected in COVID+/ARDS+ compared to COVID–/ARDS– lung specimens. More important to this analysis, COVID–/ARDS– lungs demonstrated a balanced distribution of the CD55 protein directly on the surface of the alveolar walls (Figure 2A,B). On the contrary, the CD55 protein signal in COVID+/ARDS+ lung tissue was found non-homogenously distributed and often detached from the alveolar lining, localized more likely in the lumen of the narrowed alveolar spaces (Figure 2C,D). COVID–/ARDS+ lung specimens showed lower signals of CD55 expression (Figure 2E,F) in comparison to the control group. The validity of the staining was demonstrated by the negative control image (Figure 2G). The Supplementary Image provides the intensity of the CD55 protein measured in individual specimens (Supplementary Image).

**Figure 3:**
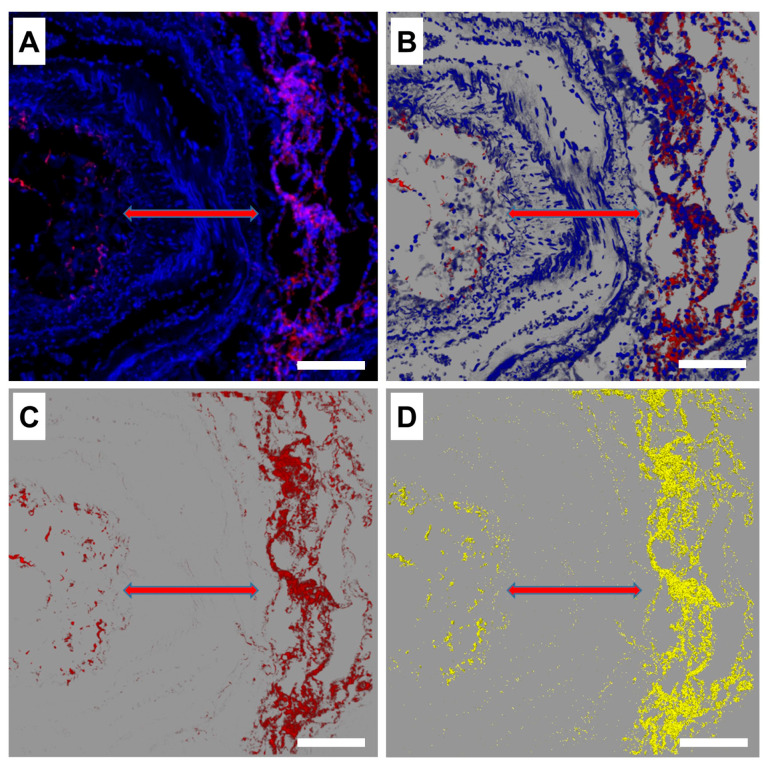
(**A**) A representative overlay immunofluorescence image of COVID–/ARDS– lung tissue containing a vessel with circa 200 µm thick muscle layer (double-headed red arrow). Red (Cy3) = CD55, blue (DAPI) = cell nuclei. (**B**) Image A in the processing sequence of the LASX software analysis tool (Version 3.5.7.23225) containing blue as well as red channels, (**C**) single red channel extraction from image B, (**D**) a binary image derived from image C. Scalebar 100 µm.

In addition, as an interesting finding, we detected the smooth muscles in blood vessels, which showed an almost negligible signal of CD55 protein compared to the surrounding alveolar tissue (Figure 2A and Figure 3A–D). Extracting the red channel from the original image (Figure 3C) and its binary image (Figure 3C) could provide a better visualization of the almost non-existent signal of CD55 protein in the area of the smooth muscles in a large vessel. Also, a weaker CD55 signal was observed in the alveolar macrophages in comparison to the signal from the alveolar lining (Figure 2B).

## 4. Discussion

The histopathology of COVID-19 lung is mainly characterized by diffuse alveolar damage, which reflects the pattern of an acute lung injury [22]. However, the histological features of diffuse alveolar damage can vary in location, i.e., focal or global change in the lung as well as in the course of ARDS, i.e., acute (exudative) phase, the organizing (proliferative) phase and the final fibrotic phase [22,23]. The early phase demonstrates alveolar edema and enlargement of the interstitium. This results in a lengthening of the blood-air diffusion path, which causes dyspnea in the affected individuals. The incorporation of diffuse or focal hyaline membranes on the surface of the alveolar sac is an important histopathological feature that is frequently found in many lethal COVID-19 cases [22]. A summary report of autopsy findings reported that 76% of the COVID-19 patients’ cases showed exudative diffuse alveolar damage (DAD), whereas 38% presented a proliferative DAD [24]. Hematoxylin eosin staining demonstrated clearly a loss of alveolar wall integrity in COVID+/ARDS+ as well as COVID–/ARDS+ lung specimens (Figure 1A). The alveolar walls were clearly thickened with focal hyperfibrotic changes, demonstrating the image of diffuse alveolar damage. COVID+/ARDS+ lung specimens displayed a massive filling of the alveolar spaces with cell debris as well as amorphous substances.

The collagen and elastic fibers provide, respectively, rigidity and elasticity to the alveolar walls in lung tissue. These fibers are embedded in a proteoglycan matrix formed by GAG, which is covalently bound to protein cores [25]. Negatively charged GAG are stained blue in the alcian blue staining. In our study, the lung tissues without alveolar damage COVID–/ARDS– showed strong homogeneous expression of GAG on both the alveolar and vessel walls. A clear, homogeneous distribution of these GAGs ensures that the alveolar walls are particularly robust. In contrast, the ARDS-affected lungs showed weaker expression of GAG. Analogously to the alcian blue staining, homogeneous and strongly stained elastic and collagen fibers arranged on the alveolar walls, as well as vascular walls, were observed in the COVID–/ARDS– lungs in the Elastica van Gieson and Masson-Goldner staining, respectively (Figure 1C,D). Correspondingly, disintegration of the fibrous tissue could be detected on the alveolar walls in the ARDS-affected lung samples. The mentioned staining in the case of COVID+/ARDS+ confirms the fibrosis of the interstitium, which reflects the fibroproliferative status of the lung tissue, as discussed before.

In this study, we were able to visualize CD55 proteins in the examined lung specimens via immunofluorescence staining. The intensity displayed by the CD55 in the images was measured and analyzed. The healthy lung tissues COVID–/ARDS– showed a significantly higher expression of the complement regulatory protein CD55 compared to the lungs affected by ARDS alone COVID–/ARDS+ (Figure 2). CD55 protein signal in COVID+/ARDS+ lung tissue was found non-homogenously distributed and often detached from the alveolar lining, also localized in the lumen of the narrowed alveolar spaces. Nevertheless, a slight decrease, however non-significant, in the CD55 expression, was seen. The collapse of the alveolar linings, as well as the accumulation of the cellular components in the alveolar spaces, adds to the total measured intensity of the analyzed CD55 protein, hence making the analysis of the protein more challenging. Overall, in our study, a lower CD55 expression in COVID–/ARDS+ or COVID+/ARDS+ lung tissue could be related to the effect of hypoxia, which can be caused by ARDS [17]. A recent study showed that RNAi-mediated CD55 silencing exacerbated bleomycin-induced lung injury, hence emphasizing the protective role of CD55 in the lung tissue [26]. In the same research, primary normal human alveolar type II epithelial cells (hAECs) purified from six normal and five idiopathic pulmonary fibrosis lungs were immunoblotted with antibodies recognizing CD55. The result showed diminished cellular CD55 expression in fibrotic lungs accompanied by CD55 fragmentation, complement dysregulation, and endoplasmatic reticulum stress.

Immunohistochemical staining analysis in a recent study by Ge, X. et al., 2023, showed elevated expression of CD55 and CD59, but not CD46, in COVID-19-infected lungs, compared to normal lungs [27]. However, only lung specimens from two female patients were used for the analysis in that study. It is known that CD55 is exploited as an attachment molecule by bacterial and viral pathogens to evade the host complement system [28,29]. Various viruses can incorporate CD55 to limit the antiviral effects of complement, also showing increased levels of cell surface CD55 in infected cells [29,30,31,32]. However, it is not yet known if this phenomenon applies to SARS-CoV-2. Additionally, during acute SARS-CoV-2 infection, CD55 was upregulated in patient blood monocytes compared to healthy control blood cells [16]. In general terms, CD55 could represent a potential therapeutic target to regulate the infection rate of such viruses in the future.

Our study, however, showed contrary results, where we observed a reduction in CD55 expression in diseased lung specimens. As mentioned before, the low CD55 expression in COVID–/ARDS+, as well as COVID+/ARDS+, can be related to the hypoxia of the alveolar cells [17]. In a bioinformatics study, a total of 324 differentially expressed genes were studied, showing a CD55 gene downregulation in SARS-CoV-2-infected samples compared to normal control samples [33]. As we know, CD55, in general, regulates the hyperactivity of the complement cascade [15]. A limited regulation could, therefore, mean higher anaphylatoxin production and higher inflammation of the lung tissue. Higher anaphylatoxin C3a and C5a mediate the down-regulation of CD46 and CD55 in the primary normal human small-airway epithelium [34]. SARS-CoV-2–infected 3-dimensional cultures secreted significantly higher levels of C3a and the proinflammatory cytokines IL-6, monocyte chemoattractant protein 1, IL-1α, and RANTES (=Regulated And Normal T cell Expressed and Secreted) [35]. In addition to exaggerated intracellular complement activation, the SARS-CoV-2 infection resulted in the destruction of the epithelial integrity in monolayer cultures of primary human airway cells as well as highly differentiated, pseudostratified, mucus-producing, ciliated respiratory tissue models [35]. Through our research, we have come to understand that both the measured intensity and the distribution of the CD55 protein in the alveolar lining with maintained integrity must be considered to evaluate CD55 expression and its relation to lung diseases.

Additionally, we observed that CD55 protein expression could not be well detected in the smooth muscles of the lung blood vessels (Figure 3). This finding is in accordance with the results of the human protein atlas (https://www.proteinatlas.org/ENSG00000196352-CD55/tissue/smooth+muscle – accessed on 5 July 2024), which presents either low or no immunohistological staining of CD55 in the smooth muscles, thus verifying the results of our immunofluorescence staining.

Age, co-morbidities, and therapeutic measures such as mechanical ventilation, medications, etc., could be influential in the complement expression of the lungs and vasculature in multiple organs; therefore, they have to be studied in relation to COVID-19 or ARDS-related outcomes. In our research, we highlighted the importance of structural integrity and CD55 protein expression in healthy lung tissue compared to diseased lungs. A major limitation of this study is the small sample size and the low power of the hypothesis test. Therefore, more research with a higher number of cohorts, as well as additional assays for the verification, is needed in the future to strengthen the validity of our results. A good understanding of the immunoregulatory complement protein CD55 expression in diseases could help in using CD55 as a biomarker for diagnosis as well as for treatment of lung diseases [20]. In general, complement interventions could play a major role in the protection of alveolar tissue during distress caused by infection or trauma in the future [18,36].

## 5. Conclusions

This study could highlight that the healthy lung tissues without ARDS-related alveolar damage COVID-/ARDS- show both stronger and homogeneous expression of GAG and compactly arranged, elastic as well as collagen fibers in the alveolar and vessel walls in comparison to COVID+/ARDS+ and COVID–/ARDS+ lung specimens. Furthermore, COVID–/ARDS– lung tissues showed higher and homogenously distributed CD55 protein expression on the alveolar walls in comparison to the disrupted COVID–/ARDS+ lung tissues. Interestingly, no significant change in CD55 immunoreactivity in the COVID+/ARDS+ lung tissue specimen was detected compared to the COVID–/ARDS– lung tissue despite ARDS. The collapse of the alveolar linings, as well as the accumulation of the cellular infiltrates, could falsely add to the total measured intensity of the CD55 protein. Therefore, we suggest that not only the measured intensity but also the distribution pattern of the CD55 protein in the alveolar lining has to be considered to evaluate the CD55 expression and its relation to the disease.

## Figures and Tables

**Table 1:** Patients’ lung specimens categorized into 3 groups (additional Supplementary Image).

Category	1	2	3
COVID-19	(−)	(+)	(−)
ARDS	(−)	(+)	(+)
Patients	n = 4	n = 5	n = 3
Sex	3M, 1F	4M, 1F	1M, 2F
Age (in years)	53–84	39–91	31–67

ARDS = Acute Respiratory Distress Syndrome; COVID = Corona Virus Disease 2019; M = male, F = Female.

## Data Availability

The data presented in this study are available in this article.

## Supplementary Materials

The following supporting information can be downloaded at: https://www.mdpi.com/article/10.3390/life14091058/s1.

## Abbreviations

ARDS	Acute respiratory distress syndrome
COVID-19	Corona Virus Disease 2019
DAD	Diffuse Alveolar Damage
DAPI	4′,6-Diamidin-2-phenylindol
GAG	Glycosaminoglycan
IL	Interleukin
MAC	Membrane attacking complex
PBS	Phosphate-buffered saline
PFA	Paraformaldehyde solution
RT	Room temperature
RNAi	RNA interference
SARS-CoV-2	Severe acute respiratory syndrome coronavirus type 2
