# Supplementary figures and images for: A Histological Analysis and Detection of Complement Regulatory Protein CD55 in SARS-CoV-2 Infected Lungs
Source: Life (Basel). 2024 Aug 23;14(9):1058. doi: 10.3390/life14091058 (PMC11432792; doi:10.3390/life14091058)

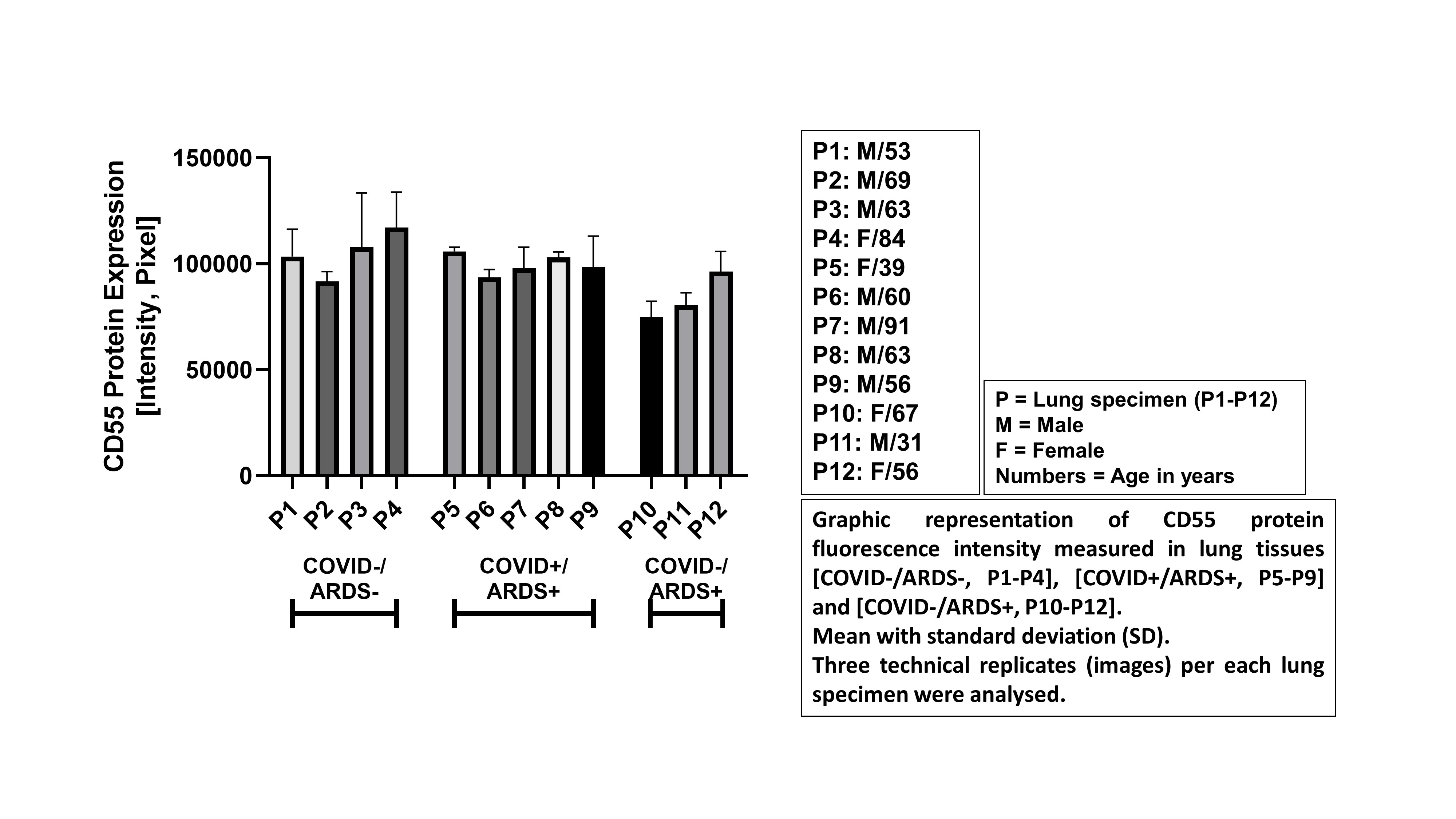

Supplement: Supplementary file 1 [file life-14-01058-s001.zip › life-3117164-supplementary.jpg]
